# Exploring knowledge, attitudes, and practices related to alcohol in Mongolia: a national population-based survey

**DOI:** 10.1186/1471-2458-13-178

**Published:** 2013-02-27

**Authors:** Alessandro R Demaio, Otgontuya Dugee, Maximillian de Courten, Ib C Bygbjerg, Palam Enkhtuya, Dan W Meyrowitsch

**Affiliations:** 1 Copenhagen School of Global Health, University of Copenhagen, PO Box 2099, Copenhagen K Dk-1014, Denmark; 2 Public Health Institute, Mongolian Ministry of Health, Olympic Street 2, Ulaanbaatar, Mongolia; 3 Section of Health Services Research, Department of Public Health, University of Copenhagen, Øster Farimagsgade 5, Copenhagen, DK-1014, Denmark

**Keywords:** Alcohol, Epidemiology, Mongolia, Asia, KAP survey, Policy, Health

## Abstract

**Background:**

The leading cause of mortality in Mongolia is Non-Communicable Disease. Alcohol is recognised by the World Health Organization as one of the four major disease drivers and so, in order to better understand and triangulate recent national burden-of-disease surveys and to inform policy responses to alcohol consumption in Mongolia, a national Knowledge, Attitudes and Practices survey was conducted. Focusing on Non-Communicable Diseases and their risk factors, this publication explores the alcohol-related findings of this national survey.

**Methods:**

A door-to-door, household-based questionnaire was conducted on 3450 people from across Mongolia. Participants were recruited using a multi-stage random cluster sampling technique, and eligibility was granted to permanent residents of households who were aged between 15 and 64 years. A nationally representative sample size was calculated, based on methodologies aligned with the WHO STEPwise approach to Surveillance.

**Results:**

Approximately 50% of males and 30% of females were found to be current drinkers of alcohol. Moreover, nine in ten respondents agreed that heavy episodic drinking of alcohol is common among Mongolians, and the harms of daily alcohol consumption were generally perceived to be high. Indeed, 90% of respondents regarded daily alcohol consumption as either ‘harmful’ or ‘very harmful’. Interestingly, morning drinking, suggestive of problematic drinking, was highest in rural men and was associated with lower-levels of education and unemployment.

**Conclusion:**

This research suggests that Mongolia faces an epidemiological challenge in addressing the burden of alcohol use and related problems. Males, rural populations and those aged 25-34 years exhibited the highest levels of risky drinking practices, while urban populations exhibit higher levels of general alcohol consumption. These findings suggest a focus and context for public health measures addressing alcohol-related harm in Mongolia.

## Background

The World Health Organization (WHO) estimates that approximately 2 billion people worldwide consume alcohol, of whom almost 80 million are believed to be diagnosable with an Alcohol-Use Disorder (Endnote A). Globally, alcohol consumption has increased in recent decades, with most of that increase occurring in Low and Middle-Income Countries (LMIC) [1,2]. Alcohol now causes 2.5 million deaths annually, representing 3.8% of the total worldwide mortality [1]. In the setting of a rising prevalence of Non-Communicable Diseases (NCDs), alcohol is recognised as one of four major NCD risk factors by WHO, and outlined as a point for mitigation following the 2011 United Nations Meeting [3,4].

However, the increase in alcohol consumption and related diseases in LMIC is likely underestimated as the majority of consumption goes unrecorded by monitoring efforts [1,2]; such as in China and India where over 40% and 75% respectively of alcohol consumed may be unacknowledged [2].

Mongolia is a lower-middle income country with a population of about 2.6 million [5]. More than 57% of Mongolians now live in urban settings as a result of rapid internal migration following transition to a free market economy in the 1990s [6,7]. Mongolia has a legal age of 18 years for purchasing alcohol, while excise tax varies depending on the type of alcoholic beverage, from less than one US dollar to six US dollars per litre. Most of the national consumption of alcohol is in the form of spirits (50%) and beer (30%), similar to its neighbour, China [8,9]. In 2011, WHO estimated that one in twenty Mongolian men had a diagnosable Alcohol-Use Disorder, while acknowledging that up to two-thirds of national alcohol consumption remains unrecorded^a^[1,8].

Alcohol has a strong presence in Mongolian culture, predating, but exacerbated by Soviet rule [10]. In rural regions, families have been making traditional Mongol Arkhi or fermented horse milk liquor for many generations – and continue to do so today. Commonly consumed throughout the day, predominantly by men, this drink is associated with celebration, strength and virility. Made in traditional leather sacks in the warmth of the family ger (customary Mongolian, wool house), this form of alcohol is widespread, but availability is seasonal, allowing recurrent periods of abstinence. More recently introduced by the Russians during the period of the Soviet Union, vodka became the drink of national choice and its spread resulted in liquor being available year-round [11]. Now a major industry in Mongolia and a contributor to government revenue through excise tax, vodka is commonly branded with the names of national heroes or Mongolian regions, evoking associations of patriotism, pride and leadership [11].

In 2005 and 2009, WHO STEPwise approach to Surveillance (STEPS) was conducted in Mongolia [12], in part focusing on alcohol epidemiology. The most recent of these studies showed that almost 50% of Mongolians aged 25-54 were classified as current drinkers^b^ of alcohol. Particularly concerning was the amount of heavy episodic drinking^c^, with 35-64 year old men reporting an average consumption of 10 standard drinks per drinking occasion. Further probing revealed that over half of men aged 25-44 and one in five females of the same age engaged in heavy episodic drinking [13]. In addition, around 50% of males aged 25-54 had consumed four or more drinks on a single occasion at least once during the previous month.

However, the STEPS surveys have limitations; while they accurately assess disease prevalence and risk factor burden, they do not assess the knowledge, attitudes or detailed practices with regards to alcohol consumption. Hence, STEPS surveys cannot inform policy-makers on the population’s level of awareness of the risks associated with heavy episodic drinking, or on the reasons for engaging in such patterns of alcohol consumption. For example, STEPS surveys cannot indicate if the majority of heavy consumption follows receiving income, and therefore cannot guide policies and regulations around pricing that may affect consumption behaviours [14].

Given these limitations of STEPS surveys, a national Knowledge, Attitudes and Practices (KAP) survey was designed and implemented in Mongolia in 2010 to explore these issues in more detail.

This KAP survey aimed to explore five domains:

1. Prevalence of alcohol consumption.

2. Prevalence of risky drinking practices among Mongolians.

3. Risk perceptions towards daily alcohol consumption.

4. Awareness of heavy episodic drinking as a public health concern for Mongolia.

5. Major social, cultural and psychosocial drivers of heavy episodic drinking.

## Methods

### Setting and population

A door-to-door, household-based questionnaire was conducted on a nationally-representative sample. The full study protocol has been published [15].

### Questionnaire

Forty-five local, trained field workers from the National Institute for Public Health administered the questionnaire, which explored a range of NCD and risk factor domains, including alcohol. Of the total 106 questions posed, 11 explored alcohol epidemiology. The wider questionnaire also included:

- Administrative demographic and geographic questions

- Risk factor specific sections covering broad NCD risk factor awareness

- Focused sections investigating public knowledge and attitudes towards specific diseases.

### Definitions of the KAP survey domains

#### Prevalence of current drinkers of alcohol

Current drinkers of alcohol were those who reported having consumed any alcohol in the previous 12 months.

#### Prevalence of risky drinking practices

Participants who reported having consumed alcohol in the previous year were asked whether they had consumed alcohol in a morning period (between waking and midday) in the previous month. They were also asked whether they had ever felt the need to cut down their own alcohol intake.

Finally, participants who reported driving a car were asked if they had ever driven while under the influence of alcohol.

#### Risk perception

Participants perception of the health risk associated with alcohol consumption was investigated using a 4-level Likert scale mean (0/not harmful, 1/moderately-harmful, 2/harmful, 3/very-harmful).

#### Awareness of heavy episodic drinking as a public health concern

Awareness of the high prevalence of heavy episodic drinking among Mongolians was estimated using a four-level Likert scale. Participants were asked if they agreed that in general Mongolians drink large amounts of alcohol on single drinking occasions (strongly disagree, disagree, agree, strongly agree).

#### The major social, psychosocial and cultural drivers of drinking practices

Participants were asked to spontaneously list the most common reasons they consumed alcohol. Next, they were read a list of common social occasions and asked which of these would be associated with heavier drinking in Mongolian society.

### Sampling methods and sample size

Sample size was calculated to obtain a nationally-representative sample and to allow disaggregated data analysis. The calculation of the nationally-representative sample was estimated considering a 95% confidence level. A design effect was adopted, based on methodologies used for the WHO STEPS. Accounting for an assumed non-response rate of 10%, final sample size was estimated at 3,854.

Participants were recruited using a multi-stage cluster sampling technique across 42 locations. Sampling used proportional population to size methods for primary and secondary sampling units, followed with simple random sampling from household lists for tertiary sampling units. Finally, a Kish method was used for participant selection within households [16].

Only permanent residents of the households sampled were eligible for recruitment and had to be aged between 15 and 64 years. In total, 3540 participants completed the survey.

While this survey does not sample the same participants as the 2009 WHO STEPS survey, both methodologies are aligned, and both nationally-representative samples are from the same broader population.

### Quality control

In order to maximise scientific rigour in this research, a number of processes were adopted in the questionnaire methodologies [15]. These included:

1. Translation and back translation

2. Peer and expert review

3. Pretesting using a Cognitive Interviewing [17]

4. Piloting.

### Analysis

Analysis was undertaken using the statistical software package SPSS (IBM SPSS 20.0.0 Statistics). Sampling error, which could potentially affect the accuracy of this survey’s results, was estimated from the standard error of variables computed taking the cluster sampling technique into account.

Multiple regression models were constructed to explore the relationship between knowledge, risk attitudes and disease or demographic parameters comparing means from Likert scale measures. Binary outcome or categorical variable questions were analysed using logistic regression expressed as bivariate or multivariate (adjusted) odds ratios (OR). Co-variables included in multivariate analysis were gender, rurality, age, education and employment.

### Limitations of methods

It should be recognised that the findings of this KAP survey may under-estimate the true burden of alcohol-related behaviours due to recall and observation bias [18,19]. In addition, while minimised through the use of local, Mongolian-speaking interviewers, participants may have felt inclined to conceal or minimise unhealthy behaviours (courtesy bias) given interviewers were, in general, doctors or government workers. This was minimised through the use of a strict questionnaire script, in which concepts were explained and questions posed in an identical fashion for each participant.

This survey adapted the CAGE clinical tool for use in a population-based survey. The CAGE questionnaire is used for the screening and diagnosis of alcohol related disorders, with an estimated sensitivity of 70% [20,21]. While this has not been formally validated in a Mongolian population, it is used widely by Mongolian clinicians and accepted as a clinical tool in Mongolian healthcare. Two CAGE questions, which combined constitute the diagnosis of the disorder, were translated and back translated by Mongolian doctors with experience in the use of this tool, as well as pre-tested and piloted on non-medically-trained Mongolians^a^.

Finally, recognizing the limited impact of knowledge on health behaviours [22], it must be stressed that this survey was not designed as a stand-alone epidemiological tool. It was rather designed as a KAP tool to align with, and triangulate the findings of the WHO STEPS surveys, for more accurate interpretation and policy development.

### Ethics approval

This study was conducted according to the principles of the Helsinki declaration and approved by the Mongolian National Ministry of Health’s Medical Ethical Committee on the 06 October 2010.

## Results

3450 of the sampled 3,854 participants agreed to take part in the study and were included in the analyses (89.5%). Of these participants, approximately 40% were male, while 50% were from urban areas (Additional file1: Table S1).

The median overall age of participants was 33 years. No significant difference was found between the median age of rural and urban participants (32 and 34 years respectively, p = 0.08), Female median age was 3 years older than male counterparts (p <0.05).

Approximately two-thirds of participants were educated to a secondary school level with 30% having been to university, 6% having received only a primary school education, and 21% being students, of which half were women. Significantly more urban participants had received secondary or tertiary education, with 2% having received only primary education in urban areas and 11% in rural areas.

By employment status, two-fifths of participants reported to be employed and 15% unemployed. One-fifth of participants were retired or home-makers, of which 75% were women. Finally, there were no significant differences in relation to employment status between rural and urban populations.

### Domain one: prevalence of alcohol consumption

This survey reveals that approximately 50% of males and 30% of females were current drinkers of alcohol (MOR 3.6), confirming the findings of the 2009 STEPS (Additional file 2: Table S2) [13].

Disaggregated by age, the highest alcohol consumption rates were observed among those 25-34 years at 52% (MOR 2.1). The occurrence of youth drinking (15-24 years) was lower than in older age groups, with approximately one in five younger Mongolians drinking alcohol. Interestingly, drinking rates in the youngest urban populations were almost twice as high as compared to rural counterparts. Indeed, 34.8% (28.8-40.8) of males and 24.9% (20.0-30.0) of females were current drinkers, as compared to 19.5% (14.7-24.3) of males and 11.0% (7.5-14.5) of females in this age group in rural areas (data not shown).

### Domain two: prevalence of risky drinking practices among Mongolians

Following, current drinkers were questioned on risky drinking practices. Questions based on the CAGE clinical assessment tool probed alcohol consumption in the morning, and explored the participants’ own perceptions around their need to reduce their alcohol intake [20,21] (Additional file 3: Table S3). Other questions aimed to identify the prevalence of current drinkers having driven a car while under the influence of alcohol.

With an increase in road traffic accidents in Mongolia [13], participants who drive a vehicle were asked whether they had ever driven under the influence of alcohol (Additional file 3: Table S3 and Additional file 4: Table S4). As a result, 15% of current drinkers who drive a vehicle reported having driven while influenced by alcohol, with one in five males and around one in thirteen females (MOR 4.1) acknowledging this behavior. No differences were found by location, education, employment or age (Additional file 4: Table S4).

To further gauge the level of risky practices, and reflecting the CAGE clinical tool for assessing problematic alcohol consumption, current drinkers were asked two additional questions: whether they had consumed alcohol between waking in the morning and lunchtime in the past month, and whether they had considered the need to reduce their own alcohol intake (Additional file 5: Table S5) [20].

Among males, almost one-third of current drinkers reported morning drinking in the past month, compared with one in eleven women (p < 0.01). When questioned about their need to reduce their alcohol intake, 24% of all males and 6% of females current drinkers reported both morning drinking and the perception that they needed to cut down their alcohol intake (MOR 5.0, p < 0.01).

Comparing urban and rural men, morning drinking was more prevalent among the former at 25.0% (21.3 - 28.7) and 16.2% (13.6 - 18.6) respectively (p < 0.05), and whilst highest in the oldest age group (25%, 18.7 – 31.3), the 25-34 year age group was most at-risk when other variables were controlled (MOR 2.0, P = 0.04).

### Domain three: risk perceptions of the general public towards daily drinking

The harms of daily alcohol consumption were generally perceived to be high among participants of this survey. More than 90% of respondents stated that they regard daily alcohol consumption as either ‘harmful’ or ‘very-harmful’ to health (data not shown).

There was no significant difference in mean perceived risk by age, education level or sex; nevertheless, urban dwellers on average regarded daily drinking to be more harmful than their rural counterparts. This urban-rural difference in perceived risk of daily drinking was small, but significant (harmful with a mean of 2.0 and moderately-harmful of 1.8 respectively, p < 0.01).

Finally, exploring the relationship between risk perception and risky drinking practices (represented by morning drinking), current drinkers who felt daily alcohol drinking was slightly harmful, as compared with very harmful, were more likely to engage in risky drinking practices (MOR 3.7, 1.1 – 12.3) (Additional file 6: Table S6).

### Domain four: awareness of heavy episodic drinking as a public health concern

Nine out of ten respondents either agreed or strongly agreed that heavy episodic drinking of alcohol is common among Mongolians.

There was no significant difference in awareness for men and women or across age groups (Additional file 7: Table S7). However, higher levels of education, being retired and urban dwelling were significantly associated with heightened levels of awareness (p < 0.05).

### Domain five: the major social, cultural and psychosocial drivers of heavy episodic drinking

‘Celebrations’ (91%, 89.5 - 92.2) as well as ‘Drinking with friends or family’ (87.1%, 85.4 – 88.3) were the most common reason for engaging in heavy episodic drinking among respondents. There were no significant differences between men and women (Additional file 8: Table S8).

Rural Mongolians were more likely than their urban counterparts to drink following receiving income at 51.6% (49.2 – 54.0) and 47.5% (45.1 – 49.1) respectively, while urban Mongolians tended to more commonly do so ‘with friends or family’ and during ‘celebrations’.

In terms of age, youth (15-24 years) were most likely to cite ‘customs or traditions’ as the reason for engaging in heavy episodic drinking, as volunteered by 67.1% (64.3 – 69.9) of these participants.

Participants were also asked, in an unprompted fashion, to list commonly used methods for psychological stress management in their everyday life (Additional file 9: Table S9). Common answers included exercise and talking with friends. Remarkably, almost one in ten men listed ‘alcohol’, while less than one in one hundred women did so (MOR 20). Furthermore, urban participants were almost three-times more likely to list alcohol as a stress reduction technique than their rural counterparts (MOR 2.6). Finally, participants aged 35 – 44 years (MOR 3.3) and those unemployed (OR 2.8) were also more likely to list alcohol use as a coping mechanism for psychological stress.

## Discussion

This KAP survey reflects and builds on existing STEPS findings, confirming high levels of alcohol consumption in Mongolia. Indeed, up to 80% of urban Mongolian men were found to have consumed alcohol in the previous year, compared to 70% and 20% of men in China and India respectively [9,23]. Contrasting the rural and urban settings, alcohol consumption was higher among urban dwellers, with up to twice as many drinkers as compared to the rural population. This finding could support previous research suggesting that high alcohol intake is linked to social and epidemiological transition processes, such as urbanisation [10]. It could be inferred that as Mongolians become wealthier, more urbanized and have continuous access to alcohol, consumption rises.

Applying the findings of this research to Public Health practice, three clear populations with distinct health risks and needs emerge: rural Mongolian men; urban Mongolians; and young Mongolians (Additional file 10: Table S10).

This national KAPS survey revealed a high level of health knowledge, particularly among urban participants, regarding the risks of daily alcohol consumption and awareness about the heavy episodic drinking prevalence in Mongolia. This is also the group with the highest prevalence of such behaviours. Combined, these findings support the notion that high levels of awareness alone may have little impact on healthy-behavioural outcomes in a population, findings which are echoed in other populations globally [22]. In addition, drink driving was common among Mongolian car-owners, more than 70% of which live in urban settings. Indeed, among current drinkers, 15% reported to have driven while influenced by alcohol; a figure approximately four times the level of the USA [24]. For Mongolia, where road-traffic accidents are now the fourth-leading cause of mortality, this represents a large but modifiable potential contributor to public risk.

These findings support the need for legislative policy responses such as harsher blood alcohol concentration laws, closer enforcement of such laws including sobriety checkpoints, and limitations to access for alcohol, rather than solely education or public awareness campaigns in urban Mongolia [1,2,25,26].

Findings also show that urban drinking was associated with celebrations and social events. In the context of the rapid urbanisation as well as the increasing Western-style branding and marketing of alcohol in recent decades, this may represent a changing social paradigm around drinking, where advertising and marketing of alcohol promote its association with festivities and celebrations, leading to higher levels of alcohol consumption in such settings. If identified as a threat to public health in Mongolia, this association may indicate the need for more stringent regulation of alcohol advertising as is seen in other nations [27,28].

In contrast, rural participants were less aware of heavy episodic drinking as a national health issue and of the risks posed by drinking. While reporting a lower drinking prevalence, higher levels of dangerous drinking behaviours were reported. These findings were particularly evident among men. This echoes similar studies from China and India, where risky alcohol consumption is also linked to male-gender and rural dwelling [29,30]. Morning drinking more specifically was associated with male-gender and rurality in Mongolia, with up to one in three rural men reporting morning drinking in the last month as current drinkers of alcohol. Although these findings may represent long-standing traditional practices in Mongolia, such as home brewing of alcohol, they also likely highlight particular health-knowledge gaps requiring targeted public health responses in rural areas. This may see regulatory, harm-reduction efforts combined with culturally-sensitive educational campaigns [14,31,32] (Additional file 10: Table S10).

Looking across the lifespan, low youth drinking rates with a peak in the late-twenties suggests that most Mongolians begin consuming alcohol during their second decade of life, a practice that peaks in middle-age. This is in contrast to many developed nations, where drinking uptake usually peaks in the teenage years [33-35], and it may help to identify a wider window of opportunity for public health efforts, for which earlier interventions are generally more effective. Such measures may include school-based harm-minimisation programs, as well as pricing and advertising regulations [26,36].

Finally, findings reveal that drinking and heavy drinking were less common among the oldest Mongolians. As alcohol in some form has been part of the Mongolian culture for many centuries, this supports the theory that the high burden of drinking in early-middle age may not be linked to the long-standing Mongolian cultural norms. Instead, a new practice, possibly associated with rapid recent socio-economic changes and urbanisation in Mongolia [7], might justify these findings. If such is true, this again supports the need for swift and meaningful public health responses as earlier outlined.

## Conclusion

In the aftermath of the 2011 High-Level Meeting on NCDs, which prioritised alcohol-harm reduction, this national survey adds to and refines existing knowledge around factors driving alcohol-related harm in Mongolia. Findings on drinking and risky-drinking prevalence, and on knowledge, risk perceptions and related attitudes towards alcohol all combine to better inform policies and programs to address this burden.

This research confirms a high prevalence of drinking among Mongolians, as well as risky drinking practices including binge drinking and morning drinking. It furthermore suggests that, in general, Mongolians hold a high level of knowledge around the harms of alcohol consumption and risky-drinking practices.

Finally, this KAP identifies three Mongolian sub-populations with distinct alcohol-related program and policy response needs, and outlines possible public health approaches for which there is an evidence-base. Indeed, despite better harm-related knowledge, urban populations exhibited higher prevalence of alcohol consumption and heavy-episodic drinking compared to rural populations. Given the speed at which Mongolia is urbanising, this may represent a growing trend and a focus for public health responses. While higher consumption prevalence was associated with urbanicity, rurality and male-gender was an independent predictor for poorer health knowledge, and was associated with morning drinking of alcohol, a predictor of chronic alcohol misuse. Finally, this research suggests that alcohol drinking peaks in the late 20s and is associated with celebrations and social activities, rendering youth a potentially important third target group for strategies on alcohol-harm reduction.

## Endnotes

^a^ Alcohol-Use Disorder is defined by DSM-5 criteria of the American Psychiatric Association, 2012.

^b^ Current drinkers are defined as participants who report having ever consumed alcohol in the previous 12 months.

^c^ For the KAPS 2009, heavy episodic drinking was 4 or more drinks in a single occasion. For the purposes of this study and article, heavy episodic drinking was redefined for lay participants as “drinking large amounts of alcohol on a single occasion”.

## Competing interests

The authors declare that they have no competing interests.

## Authors’ contributions

AD and DM drafted the manuscript. AD, OD, PA and MdC participated in the design of the study. OD obtained funding for the project. All authors read and commented on manuscript drafts. All authors read and approved the final manuscript.

## Pre-publication history

The pre-publication history for this paper can be accessed here:

http://www.biomedcentral.com/1471-2458/13/178/prepub

## Supplementary Material

Additional file 1: Table S1 Descriptive information on sample population, disaggregated by age, sex, urbanicity, educational level and employment status. Click here for file

Additional file 2: Table S2 Prevalence of current-drinkers of alcohol within the Mongolian population. Click here for file

Additional file 3: Table S3 Practices relating to risky drinking of alcohol. Click here for file

Additional file 4: Table S4 Self-reported driving while alcohol-affected among non-abstaining drivers. Click here for file

Additional file 5: Table S5 Morning drinking in the past month, and problematic drinking among current drinkers. Click here for file

Additional file 6: Table S6 Relationship between morning drinking of alcohol and risk perceptions of daily alcohol drinking. Click here for file

Additional file 7: Table S7 Awareness of heavy episodic drinking as a public health concern. Click here for file

Additional file 8: Table S8 Social and cultural drivers of heavy episodic drinking amongst Mongolians. Click here for file

Additional file 9: Table S9 Participants listing alcohol as an utilised stress reduction method. Click here for file

Additional file 10: Table S10 Translating KAP findings into public health practice. Click here for file
