# Supplementary material for: Exploring knowledge, attitudes, and practices related to alcohol in Mongolia: a national population-based survey
Source: BMC Public Health. 2013 Feb 27;13:178. doi: 10.1186/1471-2458-13-178 (PMC3606611; doi:10.1186/1471-2458-13-178)
Supplement: Additional file 1: Table S1 — Descriptive information on sample population, disaggregated by age, sex, urbanicity, educational level and employment status. [file 1471-2458-13-178-S1.doc]

Table 1 **Descriptive information on sample population, disaggregated by age, sex, urbanicity, educational level and employment status**

|  | |  | **Male** | **Female** |
| --- | --- | --- | --- | --- |
|  | | **n (% of total)** | **n (% of total)** | **n (% of total)** |
| Total |  | 3450 (100) | 1413 (42.0) | 2037 (58.0) |
| Median age |  | 33 years | 31 years | 34 years |
| Age (n=3450) | 15-24 | 1100 (28) | 506 (13.9) | 594 (14.1) |
|  | 25-34 | 721 (24.3) | 280 (12.8) | 441 (11.6) |
|  | 35-44 | 630 (23.0) | 234 (11.0) | 396 (11.9) |
|  | 45-54 | 507 (19.2) | 196 (9.3) | 311 (9.9) |
|  | 55-64 | 492 (5.5) | 197 (2.7) | 295 (2.8) |
| Location (n=3450) | Urban | 1737 (50.3) | 702 (20.3) | 1035 (29.2) |
|  | Rural | 1713 (49.7) | 711 (20.6) | 1002 (29.0) |
| Education | Primary or less | 219 (6.4) | 107 (3.1) | 112 (3.2) |
| (n= 3450) | Secondary school | 2088 (60.5) | 919 (26.6) | 1169 (33.9) |
|  | Tertiary schooling | 1143 (33.1) | 387 (11.2) | 756 (21.9) |
| Employment (n=3425) | Student | 717 (20.8) | 330 (9.6) | 387 (11.2) |
|  | Employed | 1503 (43.6) | 696 (20.2) | 807 (23.4) |
|  | Unemployed | 508 (14.7) | 204 (5.9) | 304 (8.8) |
|  | Retired/home | 697 (20.2) | 167 (4.8) | 530 (15.4) |
