# Supplementary material for: Exploring knowledge, attitudes, and practices related to alcohol in Mongolia: a national population-based survey
Source: BMC Public Health. 2013 Feb 27;13:178. doi: 10.1186/1471-2458-13-178 (PMC3606611; doi:10.1186/1471-2458-13-178)
Supplement: Additional file 2: Table S2 — Prevalence of current-drinkers of alcohol within the Mongolian population. [file 1471-2458-13-178-S2.doc]

Table 2 **Prevalence of current-drinkers of alcohol within the Mongolian population**

|  | | **Prevalence** | **CI (95**%**)** | **MOR**** | **p-value** |
| --- | --- | --- | --- | --- | --- |
| **Gender** | Female | 28.1% | (26.1 – 30) | 1.0 | - |
|  | Male | 52.7% | (50.1 – 55.3) | 3.6 (3.4 – 3.8) | <0.01 |
| **Urbanicity** | Rural | 30.5% | (28.3 – 32.7) | 1.0 | - |
|  | Urban | 45.8% | (43.5 – 48.1) | 2.0 (1.7 – 2.3) | <0.01 |
| **Age** | 15-24 | 22.0% | (19.6 – 24.5) | 1.0 | - |
|  | 25-34 | 51.9% | (48.5 – 55.6) | 2.1 (1.8 – 2.4) | <0.01 |
|  | 35-44 | 47.1% | (43.2 – 51) | 1.8 (1.5 – 2.1) | <0.01 |
|  | 45-54 | 44.2% | (39.9 – 48.5) | 1.8 (1.5 – 2.1) | <0.01 |
|  | 55-64 | 36.6% | (32.3 – 40.9) | 1.4 (1.1-1.7) | 0.04 |
| **Education** | Primary or less | 26.0% | (20.2 – 31.8) | 1.0 | - |
|  | Secondary School | 33.3% | (31.5 – 35.2) | 2.0 (1.6 – 2.4) | <0.01 |
|  | Tertiary Schooling | 49.6% | 46.7 – 52.5) | 2.6 (2.2 – 3.0) | <0.01 |
| **Employment** | Student | 15.3% | (12.7 – 17.9) | 1.0 | - |
|  | Retired/Home | 30.0% | (26.5 – 33.5) | 1.4 (1.1 – 1.7) | 0.04 |
|  | Unemployed | 36.0% | (32.1 – 39.9) | 1.5 (1.2 – 1.8) | 0.02 |
|  | Employed | 52.2% | (49.7 – 54.7) | 3.8 (3.5 – 4.1) | <0.01 |

**Multivariate Odds Ratio (MOR) adjusted for gender, urbanicity, age, educational background and employment status.
