# Supplementary material for: Exploring knowledge, attitudes, and practices related to alcohol in Mongolia: a national population-based survey
Source: BMC Public Health. 2013 Feb 27;13:178. doi: 10.1186/1471-2458-13-178 (PMC3606611; doi:10.1186/1471-2458-13-178)
Supplement: Additional file 3: Table S3 — Practices relating to risky drinking of alcohol. [file 1471-2458-13-178-S3.doc]

Table 3 **Practices relating to risky drinking of alcohol**

|  |  | | **Self-reported Driving While Alcohol Affected** | | **Self-Reported Morning Drinking in Past Month** | | **Participant Has Considered Alcohol Reduction** | | **Morning Drinking and Considered Cutting Down** | |
| --- | --- | --- | --- | --- | --- | --- | --- | --- | --- | --- |
|  |  | | % | CI (95%) | % | CI (95%) | % | CI (95%) | % | CI (95%) |
| **Gender** | | Male | 18.8% | (15.8 - 28.8) | 28.1% | (24.9 - 31.3) | 74.1% | (71.0 - 77.2) | 24.0% | (21.0 - 27.0) |
|  |  | Female | 6.6% | (4.0 - 10.0) | 8.9% | (6.5 - 11.2) | 44.2% | (39.9 - 48) | 5.9% | (4.0 - 7.8) |
| **Urbanicity** | | Urban | 16.0% | (12.9-19.0) | 16.2% | (13.6 - 18.6) | 54.8% | (51.3 - 58.3) | 13.2% | (10.9-15.5) |
|  |  | Rural | 14.3% | (10.9 - 17.7) | 25.0% | (21.3 - 28.7) | 70.7% | (66.8 - 74.6) | 20.7% | (17.2 - 24.2) |
| **Age** | | 15-24* | - | - | 12.3% | (8.1 - 16.4) | 50.6% | (44.3 - 56.9) | 9.5% | (5.8 - 13.2) |
|  |  | 25-34 | 20.2% | (15.4 - 25.0) | 20.1% | (16.0 - 24.3) | 61.4% | (56.5 - 66.3) | 16.0% | (12.3 - 19.7) |
|  |  | 35-44 | 15.3% | (10.5 - 20.1) | 22.2% | (17.5 - 27.0) | 63.3% | (57.8 - 68.8) | 18.5% | (14.1 - 22.9) |
|  |  | 45-54 | 18.4% | (12.1 - 24.7) | 19.6% | (14.4 - 24.8) | 68.3% | (62.2 - 74.4) | 18.3% | (13.2 - 23.4) |
|  |  | 55-64 | 15.5% | (8.3 - 22.7) | 25.0% | (18.7 - 31.3) | 62.2% | (55.1 - 69.3) | 18.9% | (13.2 - 24.6) |

* Driving was not explored in non-driving, 15-24 years group.
