# Supplementary material for: Exploring knowledge, attitudes, and practices related to alcohol in Mongolia: a national population-based survey
Source: BMC Public Health. 2013 Feb 27;13:178. doi: 10.1186/1471-2458-13-178 (PMC3606611; doi:10.1186/1471-2458-13-178)
Supplement: Additional file 4: Table S4 — Self-reported driving while alcohol-affected among non-abstaining drivers. [file 1471-2458-13-178-S4.doc]

Table 4 **Self-reported driving while alcohol-affected among non-abstaining drivers**

|  | | **MOR**** | **p-value** |
| --- | --- | --- | --- |
| Gender | Female | 1.0 | - |
|  | Male | 4.1 (2.3 – 7.0) | <0.01 |
| Urbanicity | Rural | 1.0 |  |
|  | Urban | 1.2 (0.8 – 1.7) | 0.3 |
|  | 15-24* | - | - |
|  | 25-34 | 1.0 | - |
| Age | 35-44 | 0.7 (0.3 – 1.5) | 0.4 |
|  | 45-54 | 1.0 (0.4 – 2.1) | 0.5 |
|  | 55-64 | 0.8 (0.4 – 1.8) | 0.4 |
|  | Tertiary schooling | 1.0 |  |
| Education | Secondary school | 0.9 (0.3 – 2.3) | 0.3 |
|  | Primary or less | 1.6 (1.0 – 2.4) | 0.07 |
|  | Student* | - |  |
|  | Employed |  |  |
| Employment | Unemployed | 0.6 (0.3 – 1.4) | 0.3 |
|  | Retired/home | 0.6 (0.3 – 1.4) | 0.2 |

* Driving was not explored in non-driving, 15-24 years group or students.

**Multivariate Odds Ratio (MOR) adjusted for gender, urbanicity, age, educational background and employment status.
