# Supplementary material for: Exploring knowledge, attitudes, and practices related to alcohol in Mongolia: a national population-based survey
Source: BMC Public Health. 2013 Feb 27;13:178. doi: 10.1186/1471-2458-13-178 (PMC3606611; doi:10.1186/1471-2458-13-178)
Supplement: Additional file 5: Table S5 — Morning drinking in the past month, and problematic drinking among current drinkers. [file 1471-2458-13-178-S5.doc]

Table 5 **Morning drinking in the past month, and problematic drinking among current drinkers**

| **Morning drinking in the past month** | |  |  | **Morning drinking in the past month and considered reducing intake** |  |  |
| --- | --- | --- | --- | --- | --- | --- |
|  |  | MOR** | p-value |  | MOR** | p-value |
| Gender | Female | 1.0 |  | Female | 1.0 |  |
|  | Male | 4.0 (2.8 - 5.6) | <0.01 | Male | 5.0 (3.4-7.5) | <0.01 |
| Urbanicity | Urban | 1.0 |  | Rural | 1.0 |  |
|  | Rural | 1.5 (1.1 – 2.1) | 0.02 | Urban | 1.6 (1.1-2.2) | 0.03 |
| Age group | 15-24 | 1.0 |  | 15-24 | 1.0 |  |
|  | 25-34 | 2.0 (1.1 – 3.8) | 0.04 | 25-34 | 2.2 (1.2-4.0) | 0.03 |
|  | 35-44 | 1.0 (0.6 – 1.6) | 0.07 | 35-44 | 2.5 (1.3-4.6) | 0.04 |
|  | 45-54 | 0.9 (0.5 – 1.5) | 0.08 | 45-54 | 2.2 (1.2-4.3) | 0.04 |
|  | 55-64 | 1.1 (0.7 – 2.0) | 0.2 | 55-64 | 2.0 (1.0-4.1) | 0.06 |
| Schooling | Tertiary schooling | 1.0 |  | Tertiary schooling | 1.0 |  |
|  | Secondary school | 0.8 (0.4 – 1.6) | 0.2 | Secondary school | 1.0 (0.5-2.1) | 0.09 |
|  | Primary or less | 1.1 (0.8 -1.5) | 0.09 | Primary or less | 1.0 (0.5-2.3) | 0.09 |
| Employment | Student | 1.0 |  | Student | 1.0 |  |
|  | Employed | 1.2 (0.5 – 2.7) | 0.5 | Employed | 0.9 (0.4-2.0) | 0.4 |
|  | Unemployed | 1.2 (0.8 – 2.0) | 0.08 | Unemployed | 1.1 (0.5-2.7) | 0.09 |
|  | Retired/home | 0.9 (0.5 – 1.5) | 0.2 | Retired/home | 1.1 (0.4-2.5) | 0.6 |

**Multivariate Odds Ratio (MOR) adjusted for gender, urbanicity, age, educational background and employment status.
