# Supplementary material for: Exploring knowledge, attitudes, and practices related to alcohol in Mongolia: a national population-based survey
Source: BMC Public Health. 2013 Feb 27;13:178. doi: 10.1186/1471-2458-13-178 (PMC3606611; doi:10.1186/1471-2458-13-178)
Supplement: Additional file 6: Table S6 — Relationship between morning drinking of alcohol and risk perceptions of daily alcohol drinking. [file 1471-2458-13-178-S6.doc]

Table 6 **Relationship between morning drinking of alcohol and risk perceptions of daily alcohol drinking**

| **Morning drinking in the past month** | |  | |
| --- | --- | --- | --- |
|  |  | MOR** | p-value |
| **Risk perception of daily drinking** | Very harmful | 1.0 | - |
|  | Harmful | 3.7 (1.1-12.3) | 0.04 |
|  | Slightly harmful | 3.5 (1.1-11.5) | 0.04 |
|  | Not harmful* | - | - |

* No participants gave this response.

**Multivariate Odds Ratio (MOR) adjusted for gender, urbanicity, age, educational background and employment status.
