# Supplementary material for: Exploring knowledge, attitudes, and practices related to alcohol in Mongolia: a national population-based survey
Source: BMC Public Health. 2013 Feb 27;13:178. doi: 10.1186/1471-2458-13-178 (PMC3606611; doi:10.1186/1471-2458-13-178)
Supplement: Additional file 7: Table S7 — Awareness of heavy episodic drinking as a public health concern. [file 1471-2458-13-178-S7.doc]

Table 7**Awareness of heavy episodic drinking as a public health concern**

|  | | **MOR**** | **p-value** |
| --- | --- | --- | --- |
| **Gender** | Female | 1.0 | - |
|  | Male | 0.9 (0.6 – 1.2) | 0.3 |
| **Urbanicity** | Rural | 1.0 | - |
|  | Urban | 2.2 (1.8 – 2.4) | <0.01 |
| **Age** | 15-24 | 1.0 | - |
|  | 25-34 | 1.0 (0.7 – 1.3) | 0.07 |
|  | 35-44 | 0.9 (0.6 – 1.3) | 0.1 |
|  | 45-54 | 0.8 (0.5 – 1.3) | 0.09 |
|  | 55-64 | 0.8 (0.6 – 1.2) | 0.09 |
|  | Primary or less | 1.0 | - |
| **Education** | Secondary school | 2.0 (1.7 – 2.3) | <0.01 |
|  | Tertiary schooling | 3.0 (2.6 – 3.4) | <0.01 |
|  | Student | 1.0 | - |
|  | Retired/home | 2.0 (1.7 – 2.3) | 0.05 |
| **Employment** | Unemployed | 1.2 (0.8 – 1.6) | 0.4 |
|  | Employed | 1.3 (0.9 – 1.7) | 0.2 |

**Multivariate Odds Ratio (MOR) adjusted for gender, urbanicity, age, educational background and employment status.
