# Supplementary material for: Exploring knowledge, attitudes, and practices related to alcohol in Mongolia: a national population-based survey
Source: BMC Public Health. 2013 Feb 27;13:178. doi: 10.1186/1471-2458-13-178 (PMC3606611; doi:10.1186/1471-2458-13-178)
Supplement: Additional file 8: Table S8 — Social and cultural drivers of heavy episodic drinking amongst Mongolians. [file 1471-2458-13-178-S8.doc]

**Table 8** Social and cultural drivers of heavy episodic drinking amongst Mongolians

|  | **After receiving your income** | | | **Celebrations** | | **Drinking with friends or family** | | **Customs or Traditions** | |
| --- | --- | --- | --- | --- | --- | --- | --- | --- | --- |
|  | % | | CI (95% | % | CI (95% | % | CI (95% | % | CI (95% |
|  | 49.5% | | (47.1 – 51.9) | 91.0% | (89.5 – 92.2) | 87.1% | (85.4 – 88.3) | 60.8% | (58.6 – 63.1) |
| **Male** | 48.5% | | (45.9 - 51.1) | 90.7% | (89.2 - 92.2) | 88.4% | (86.7 - 90.1) | 60.9% | (58.4 - 63.4) |
| **Female** | 50.3% | | (48.1 - 52.5) | 91.1% | (89.9 - 92.3) | 85.0% | (83.4 - 86.5) | 60.8% | (58.7 - 63) |
|  |  | | | | | | | | |
| **Urban** | 47.5% | (45.1 - 49.1) | | 93.5% | (92.3 - 94.7) | 89.8% | (88.4 - 91.2) | 59.0% | (56.7 - 61.3) |
| **Rural** | 51.6% | (49.2 - 54) | | 88.3% | (86.6 - 89.8) | 82.9% | (81.1 - 84.7) | 62.8% | (60.5 - 65.1) |
| **Age:** |  | | | | | | | | |
| **15-24** | 51.2% | (48.3 - 54.1) | | 91.4% | (89.7 - 93) | 84.9% | (82.8 - 87) | 67.1% | (64.3 - 69.9) |
| **25-34** | 46.5% | (48.9 - 50.1) | | 90.4% | (88.2 - 92.5) | 86.7% | (84.2 - 89.2) | 58.7% | (55.1 - 62.3) |
| **35-44** | 43.3% | (39.4 - 47.2) | | 89.8% | (87.4 - 92.2) | 86.2% | (83.5 - 88.9) | 54.6% | (50.7 - 58.5) |
| **45-54** | 48.9% | (44.9 - 53.2) | | 89.7% | (87 - 92.3) | 87.2% | (84.3 - 90.1) | 55.6% | (51.3 - 59.9) |
| **55-64** | 58.9% | (54.6 - 63.2) | | 93.3% | (91.1 - 95.5) | 88.6% | (85.8 - 91.4) | 63.6% | (59.6 - 67.8) |
