# Supplementary material for: Exploring knowledge, attitudes, and practices related to alcohol in Mongolia: a national population-based survey
Source: BMC Public Health. 2013 Feb 27;13:178. doi: 10.1186/1471-2458-13-178 (PMC3606611; doi:10.1186/1471-2458-13-178)
Supplement: Additional file 9: Table S9 — Participants listing alcohol as an utilised stress reduction method. [file 1471-2458-13-178-S9.doc]

Table 9 **Participants listing alcohol as an utilised stress reduction method**

|  | | **MOR**** | **p-value** |
| --- | --- | --- | --- |
| **Gender** | Female | 1.0 |  |
|  | Male | 20 (10.1 – 39.7) | <0.01 |
| **Urbanicity** | Rural | 1.0 |  |
|  | Urban | 2.6 (1.6 – 4.2) | 0.02 |
|  | 15-24 | 1.0 |  |
|  | 25-34 | 1.6 (0.8 - 3.6) | 0.4 |
| **Age** | 35-44 | 3.3 (1.6 - 7.1) | 0.03 |
|  | 45-54 | 2.0 (0.9 - 4.6) | 0.07 |
|  | 55-64 | 1.4 (0.6 - 3.7) | 0.08 |
|  | Tertiary schooling | 1.0 |  |
| **Education** | Secondary school | 1.5 (0.4 – 5.0) | 0.09 |
|  | Primary or less | 2.5 (0.7 – 8.6) | 0.08 |
|  | Student | 1.0 |  |
|  | Employed | 1.7 (0.7 - 3.7) | 0.08 |
| **Employment** | Unemployed | 2.8 (1.1 - 5.0) | 0.04 |
|  | Retired/home | 2.9 (0.9 – 6.2) | 0.07 |

**Multivariate Odds Ratio (MOR) adjusted for gender, urbanicity, age, educational background and employment status.
