# Supplementary material for: Exploring knowledge, attitudes, and practices related to alcohol in Mongolia: a national population-based survey
Source: BMC Public Health. 2013 Feb 27;13:178. doi: 10.1186/1471-2458-13-178 (PMC3606611; doi:10.1186/1471-2458-13-178)
Supplement: Additional file 10: Table S10 — Translating KAP findings into public health practice. [file 1471-2458-13-178-S10.doc]

Table 10 **Translating KAP findings into public health practice**

| **At-risk population** | **New findings from KAP study** | **Suggestions for public health practice** |
| --- | --- | --- |
| Rural Mongolian men | High prevalence of morning drinking; lower levels of population health knowledge. | Educational programs reflecting rural cultural practices; incentives to reduce morning drinking. |
| Urban Mongolians | Higher drinking prevalence, linked to social activities; higher consumption in younger Mongolians; concentration of drink driving. | Introduction of harsher blood alcohol concentration laws; closer enforcement of legal drinking age laws; regulatory measures including taxation; limitations around advertising and the positive portrayal of alcohol. |
| Young Mongolians | Low drinking in youth and a peak in 20s/30s; heavy drinking most common in urban, early-middle-aged males; drinking linked to social activity & celebrations. | Targeted prevention programs; tightening of age-limit enforcement; increase in price-based harm-reduction; limitations around advertising and the positive portrayal of alcohol to youth. |
